# Trends of Cardiovascular Interventions Stratified by Body Mass Index: An Analysis of the 2016-2020 Nationwide Inpatient Sample Population

**DOI:** 10.7759/cureus.38550

**Published:** 2023-05-04

**Authors:** Endurance O Evbayekha, Henry O Aiwuyo, Jessica C Obonna, Okelue E Okobi, Jenny J Onyema, Enoobong Aderonke Adedoye, Mujeeb A Salawu, Uchechukwu O Ogbonna, Jane N Nwafor, Oluwasayo J Owolabi, Elochukwu U Nwachukwu, Chioma Ezuma-Ebong, Brume J Bekibele, Precious A Akinsanya, Theresa O Akewe

**Affiliations:** 1 Internal Medicine, St. Luke's Hospital, Chesterfield, USA; 2 Internal Medicine, Brookdale University Hospital Medical Center, Brooklyn, USA; 3 Epidemiology and Public Health, Memorial University of Newfoundland, St. John's, CAN; 4 Family Medicine, Medficient Health Systems, Laurel, USA; 5 Family Medicine, Lakeside Medical Center, Belle Glade, USA; 6 Family Medicine, Park Plaza Medical Clinic, Edmonton, CAN; 7 Internal Medicine, Mater Dei Hospital, Msida, MLT; 8 Internal Medicine and Psychiatry, Houston Health Department, Houston, USA; 9 Surgery, Barau Dikko Teaching Hospital, Kaduna, NGA; 10 Internal Medicine, University of the District of Columbia, Silver Spring, USA; 11 Psychiatry, Lugansk State Medical University, Lugansk, UKR; 12 Family Medicine, University of Uyo Teaching Hospital, Uyo, NGA; 13 Internal Medicine, Angelic Care Hospital, Abuja, NGA; 14 The Cancer Center, The Ottawa Hospital, Ottawa, CAN; 15 Oncology, Holy Name Medical Center, Teaneck, USA; 16 Family Medicine, University of Benin, Benin City, NGA; 17 Family and Community Medicine, Milk River Community Health Center, Milk River, CAN

**Keywords:** nationwide inpatient sample (nis), weight loss, cardiometabolic health, weight management, cardiovascular outcomes, cardiovascular interventions, obesity

## Abstract

Background

There is a scarcity of studies delineating the trends of cardiovascular interventions in the hospitalized population stratified by body mass index (BMI). Our study aimed to study the burden of cardiovascular interventions and outcomes by BMI.

Methods

We retrospectively analyzed the Nationwide Inpatient Sample (NIS) database between January 2016 and December 2020. We identified the population of interest using the International Classification of Diseases, Tenth Revision (ICD-10) code. We studied the BMI in five categories: "healthy weight" (HW; BMI < 19.9-24.9 kg/m2), "overweight" (OV; BMI = 25-29.9 kg/m2), "obesity class one" (OB1; BMI = 30-34.9 kg/m2), "obesity class two" (OB2; BMI = 35-39.9 kg/m2), and "obesity class three" (OB3; BMI > 40 kg/m2).

Results

There were 5,654,905 hospitalizations with an ICD-10 code related to BMI within this study period. The HW group had 1,103,659 (19.5%) hospitalizations, the OV group had 462,464 (8.2%), the OB1 group had 1,095,325 (19.4%), the OB2 group had 1,036,682 (18.3%), and the OB3 group had 1,956,775 (34.6%) hospitalizations. The mean age of the population with obesity was as follows: OB1 = 61 years (SD = 16); OB2 = 58 years (SD = 15.9); and OB3 = 55 years (SD = 15.5). The mean ages of the HW and OV groups were 68 years (SD = 16.6) and 65 years (SD = 16.1), respectively.

In the HW group, there were 948 (8.1%) hospital admissions for aortic valve replacement (AVR), 54 (11%) for aortic valve repair (AVRr), 737 (15.9%) for mitral valve replacement (MVRr), 12 (17.1%) for mitral valve repair (MVR), 79 (2.2%) for left atrial appendage (LAA) closure, and 3390 (5.2%) for percutaneous coronary intervention (PCI). The OV group had 1049 (8.9%) hospital admissions for AVRs, 42 (9%) for AVRr, 461 (10%) for MVRr, four (5.7%) for MVR, 307 (8.6%) for LAA closure, and 5703 (8.8%) for PCIs.

The OB1 group had 3326 (28.4%) hospital admissions for AVR, 125 (26.9%) for AVRr, 1229 (26.7%) for MVRr, 23 (32.9%) for MVR, 1173 (32.9%) for LAA, and 20,255 (31.3%) for PCI, while the OB2 group had 2725 (23.3%) hospital admissions for AVR, 105 (22.6%) for AVRr, 898 (19.4%) for MVRr, 11 (15.7%) for MVR, 933 (26.2%) for LAA, and 16,773 (25.9%) for PCI. Lastly, the OB3 group had 3626 (31%) hospital admissions for AVR, 139 (29.9%) for AVRr, 1285 (27.8%) for MVRr, 20 (28.6%) for MVR, 1063 (29.9%) for LAA, and 18,589 (28.7%) for PCI.

Conclusion

Our study supports the evidence of increased cardiovascular interventions with increasing BMI. Albeit, an inconsistent presentation across the spectrum of cardiovascular diseases and outcomes, for example, equal or better outcomes in obese cohorts compared to the healthy weight population undergoing PCI. However, the increasing cardiovascular intervention burden in the youngest studied population suggests a rise in the cardiovascular disease burden among the young and partially explains their better outcomes. Steps to include weight management for these patients are paramount.

## Introduction

Obesity and overweight have been hot topics on the front burner in various spheres in the last two decades and paradoxically have been on the rise despite the attention they have been receiving. Cardiovascular disease and interventions have also shared the same trajectory at the same time. An individual is regarded as overweight when their body weight (in kilograms) to height (in meters square) ratio is higher than that considered healthy but not in the "obese" zone based on classification according to the Centers for Disease Control and Prevention (CDC) [1]. In the past, the Metropolitan Life Insurance data expressed fat as a percentage of total body mass and used this for classification [2]. The World Health Organization’s definition of obesity and overweight describes these conditions as the abnormal, excessive accumulation of adipose tissue that predisposes to health risks [3]. The Obesity Medicine Association defines obesity as a "chronic, relapsing, multi-factorial, neurobehavioral disease, wherein an increase in body fat promotes adipose tissue dysfunction and abnormal fat mass physical forces, resulting in adverse metabolic, biomechanical, and psychosocial health consequences" [4].

The body mass index (BMI) is meant to be used as a screening tool for overweight and obesity but not for diagnostic purposes as it does have its limitations, such as not accounting for muscle mass, ethnic variations, and inability to distinguish between "good" vs. "bad" fat [5]. Increasingly, obesity is being recognized as a chronic disease condition. Several postulations have been made in various studies and observations on the correlation between increasing BMI and cardiovascular diseases and cardiovascular interventions [6].

Obesity creates an altered metabolic profile due to the adaptation of vital tissues and organs of the body. The capillary network surrounding the adipose tissue bed is enormous, with approximately 10% of net tissue wet weight from the abundant fluid reserves in the interstitial space of the adipose tissue. This means serious consequences for individuals with heart failure and other cardiovascular illnesses. This is often mitigated in the early stages of heart disease by the β-1 receptor vasodilation of the capillary networks around the adipocytes. Consequently, there is a decrease in perfusion per unit of the adipocytes with increasing total body fat despite a seemingly initial increase in cardiac output with total fat mass due to the inaccessibility of the fluid in the interstitium [6].

The adipocytes' extensive blood circulation makes them an extremely effective endocrine system. They synthesize leptin, adiponectin, tumor necrosis factor-α (TNF-α), interleukin-6 (IL-6), plasminogen activator inhibitor-1, resistin, lipoprotein lipase, insulin-like growth factor-I (IGF-I), angiotensinogen, fibrinogen, and C-reactive protein (CRP) [6,7]. Studies have shown that adipocytes make up 30% of total body IL-6. This is important as IL-6 is a crucial component of chronic inflammation and an important trigger for coronary heart disease and acute coronary syndromes [8].

According to the National Health and Nutrition Examination Survey (NHANES), the national trend of obesity rose from 30.5% in 1999 to 41.9% in 2020 [9]. This trajectory cost the United States an estimated $173 billion in 2019 [10]. Our study aimed at the most recent trends in the inpatient population setting utilizing the Nationwide Inpatient Sample (NIS). We sort to establish the relationship between individuals' BMI and cardiovascular interventions and outcomes in these cohorts.

## Materials and methods

Database

We retrospectively analyzed the NIS database from 2016 to 2020. We objectively selected our sample using the International Classification of Diseases, Tenth Revision (ICD-10) code. We excluded all participants without an ICD-10 code for their BMI. We defined and classified obesity based on the CDC and WHO classifications, as seen in Table 1 below. We studied the BMI in five categories: "healthy weight" (HW; BMI < 19.9-24.9 kg/m2), "overweight" (OV; BMI = 25-29.9 kg/m2), "obesity class one" (OB1; BMI = 30-34.9 kg/m2), "obesity class two" (OB2; BMI = 35-39.9 kg/m2), and "obesity class three" (OB3; BMI > 40 kg/m2).

**Table 1:** Classification of obesity according to the WHO based on BMI

BMI range	WHO (kg/m^2^)	Asian (kg/m^2^)
Underweight	≤18.5	≤18.5
Normal weight	18.5-24.9	18-22.9
Overweight	25-29.9	23-24.9
Stage I obesity	30-34.9	25-29.9
Stage II obesity	35-39.9	30-34.9
Stage III obesity	≥40	≥35

Analysis

We used SAS version 9.4 (SAS Institute Inc., Cary, NC) to conduct all analyses. We represented categorical variables as frequencies and percentages and continuous variables as means with confidence intervals (CI) or standard deviations (SD). We adopted a p-value of <0.05 as significant. We used the chi-square test to analyze the outcomes of our categorical variables and proc univariate analysis to obtain the measures of central tendency (mean, median, and mode).

## Results

**Figure 1:**
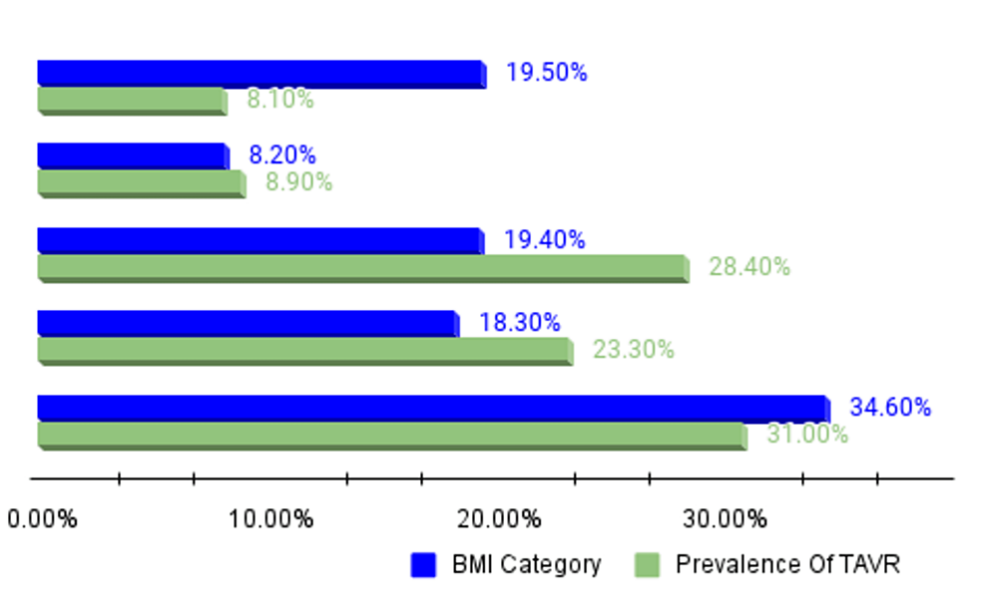
BMI classes and prevalence of TAVR within the respective classes From top to bottom of the bar chart: Healthy weight = 19.50%; TAVR prevalence = 8.10%. Overweight = 8.20%; TAVR prevalence = 8.90%. Obesity class 1 = 19.40%; TAVR prevalence = 28.40%. Obesity class 2 = 18.30%; TAVR prevalence = 23.30%. Obesity class 3 = 34.60%; TAVR prevalence = 31%. BMI = body mass index; TAVR = transcatheter aortic valve replacement.

There were 5,654,905 hospitalizations with an ICD-10 code related to BMI within this study period. The HW group had 1,103,659 (19.5%) hospitalizations, the OV group had 462,464 (8.2%), the OB1 group had 1,095,325 (19.4%), the OB2 group had 1,036,682 (18.3%), and the OB3 group had 1,956,775 (34.6%) hospitalizations. Our cardiovascular interventions of interest were percutaneous aortic valve replacement (AVR), percutaneous aortic valve repair (AVRr), percutaneous mitral valve replacement (MVRr), percutaneous mitral valve repair (MVR), and percutaneous coronary intervention (PCI). We looked at these interventions per individual BMI groups and the trend over five years. During this time, 59,348 AVR, 2465 AVRr, 21,708 MVRr, 577 MVR, and 322,262 PCI were captured in our dataset. Our cardiovascular disease outcomes of interest were Takotsubo cardiomyopathy (TAK), peripheral artery disease (PAD), coronary artery disease (CAD), ST-segment elevation myocardial infarction (STEMI), non-STEMI (NSTEMI), and atrial fibrillation (AFIB). During this time, there were 40,008 hospitalizations for TAK, 3,301,140 for PAD, 5,667,387 for CAD, 222,767 for STEMI, 719,492 for NSTEMI, and 4,204,416 for AFIB. A pictorial representation of the prevalence of AVR stratified by BMI is depicted in Figure 1.

The HW population had an overall incidence of TAK of 41.2% (3140) hospitalizations, with a gradual uptrend in incidence from 466 (14.8%) hospitalizations in 2016 to 714 (22.7%) hospitalizations in 2020 (p < 0.0001). The incidence of TAK in overweight and obesity classes 1, 2, and 3 was 701 (9.2%), 1265 (16.6%), 1077 (14.1%), and 1430 (18.8%), respectively, without changes in trend over the years. The population with a healthy weight had 133,961 (12.1%) hospitalizations for PAD, 206,521 (18.7%) had STEMI, 23,294 (15.7%) had NSTEMI, and 187,547 (20.4%) had AFIB. In the hospitalizations that have had a history of intervention, there were 948 (8.1%) hospital admissions for AVR, 54 (11%) for AVRr, 737 (15.9%) for MVRr, 12 (17.1%) for MVR, 79 (2.2%) for left atrial appendage (LAA) closure, and 3390 (5.2%) for PCI. A pictorial representation of the prevalence of AVR stratified by BMI is depicted in Figure 1.

The OV group had 701 (9.2%) admissions for TAK, 71,962 (15.6%) for PAD, 112,464 (24.3%) for CAD, 4041 (10.5%) for STEMI, 13,414 (9%) for NSTEMI, and 80,548 (8.8%) for AFIB. Cardiovascular interventions increased over the study period. There were 1049 (8.9%) admissions for AVR, 42 (9%) for AVRr, 461 (10%) for MVRr, four (5.7%) for MVR, 307 (8.6%) for LAA closure, and 5703 (8.8%) for PCIs.

The OB2 group had 1077 (14.1%) admissions for TAK, 166,620 (16.1%) admissions for PAD, 234,257 (22.6%) for CAD, 8930 (23.2%) for STEMI, 30,789 (20.8%) for NSTEMI, and 158,671 (17.3%) for AFIB. The cardiovascular intervention prevalence in this group was 2725 (23.3%) hospital admissions for AVR, 105 (22.6%) for AVRr, 898 (19.4%) for MVRr, 11 (15.7%) for MVR, 933 (26.2%) for LAA, and 16,773 (25.9%) for PCI.

The OB3 group had 1430 (18.8%) admissions for TAK, 326,183 (16.7%) admissions for PAD, 355,353 (18.2%) for CAD, 9579 (24.9%) for STEMI, 43,496 (29.3%) for NSTEMI, and 319,922 (34.8%) for AFIB. The cardiovascular intervention prevalence in this group was 3626 (31%) hospital admissions for AVR, 139 (29.9%) for AVRr, 1285 (27.8%) for MVRr, 20 (28.6%) for MVR, 1063 (29.9%) for LAA, and 18,589 (28.7%) for PCI.

With regard to PCI mortality, the HW group had a 6.61% (p < 0.0001) mortality. In comparison, the OV group had 1.77% (p = 0.08) mortality, the OB1 group had 1.42% (p < 0.0001) mortality, the OB2 group had 1.59% (p < 0.0001) mortality, and the OB3 group had a 2.29% (p = 0.08) in-hospital mortality. With regards to the population that survived PCI, their mean length of hospitalization was 7.7 days (SD = 8.8) in the HW group, 4.4 days (SD = 5.2) in the OW group, 3.5 days (SD = 3.8) in the OB1 group, 3.6 days (SD = 3.6) in the OB2 group, and 4.5 days (SD = 5.3) in the OB3 group. Table 2 below shows a breakdown of cardiovascular intervention outcomes by BMI category.

**Table 2:** Prevalence of cardiac interventions stratified according to BMI

Interventions/cardiovascular outcomes	Healthy weight = 1,103,659	Overweight = 462,464	Obesity class 1 = 1,095,325	Obesity class 2 = 1,036,682	Obesity class 3 = 1,956,775
Takotsubo cardiomyopathy (TAK)	3140 (41.2%)	701 (9.2%)	1265 (16.6%)	1077 (14.1%)	1430 (18.8%)
Peripheral artery disease (PAD)	133,961 (12.1%)	71,962 (15.6%)	177,472 (16.2%)	166,620 (16.1%)	326,183 (16.7%)
Coronary artery disease (CAD)	206,521 (18.7%)	112,464 (24.3%)	274,106 (25.0%)	234,257 (22.6%)	355,353 (18.2%)
Segment elevation myocardial infarction (STEMI)	4193 (10.9%)	4041 (10.5%)	11,655 (30.4%)	8930 (23.2%)	9579 (24.9%)
Non-ST segment elevation myocardial infarction (NSTEMI)	23,294 (15.7%)	13,414 (9%)	37,318 (25.2%)	30,789 (20.8%)	43,496 (29.3%)
Atrial fibrillation (AFIB)	187,547 (20.4%)	80,548 (8.8%)	172,501 (18.8%)	15,8671 (17.3%)	319,922 (34.8%)
Aortic valve replacement (AVR)	948 (8.1%)	1049 (8.9%)	3326 (28.4%)	2725 (23.3%)	3626 (31%)
Aortic valve repair (AVRr)	54 (11.6%)	42 (9%)	125 (26.9%)	105 (22.6%)	139 (29.9%)
Mitral valve repair (MVR)	12 (17.1%)	4 (5.7%)	23 (32.9%)	11 (15.7%)	20 (28.6%)
Mitral valve replacement (MVRr)	737 (15.9%)	461 (10%)	1229 (26.7%)	898 (19.4%)	1285 (27.8%)
Percutaneous coronary intervention (PCI)	3390 (5.2%)	5703 (8.8%)	20,255 (31.3%)	16,773 (25.9%)	18,589 (28.7%)
Left atrial appendage occlusion (LAAO)	79 (2.2%)	307 (8.6%)	1173 (32.9%)	933 (26.2%)	1063 (29.9%)

## Discussion

Much has been discussed in the literature about the obesity paradox, the inverse correlation between morbid obesity, and negative cardiovascular outcomes compared to their non-obese cohorts. In a study conducted in 2017, the morbidly obese individuals had the same short and midterm overall mortality outcomes compared to their nonobese counterparts, even though they had more intra and postprocedural complications [11,12]. Our study revealed that despite having more obese individuals undergoing cardiovascular interventions, the length of hospitalization was reduced significantly in the obese population, particularly in the OB3 group.

Some studies have shown that obese individuals with TAK have relatively higher odds of developing cardiac complications from the disease process than normal-weight individuals. They, however, were similar in terms of all-cause mortality, length of stay (LOS), and hospital charges [13]. In our study, after stratifying the population according to BMI, the LOS was shorter in obese and overweight patients who had a PCI. The LOS of the HW group with PCI was 7.7 days (SD = 8.8); it was 4.4 days (SD = 5.2) in the OV group, 3.5 days (SD = 3.8) in the OB1 group, 3.6 days (SD = 3.6) in the OB2 group, and 4.5 days (SD = 5.3) in the OB3. Interestingly, the LOS of the individuals with HW was the longest. We also saw a decreased incidence of TAK in all obese groups, including the OV group, compared to the HW group. This may suggest that obesity may paradoxically affect the incidence of TAK. This was also reported in a study by Madias et al., who reported a lower mortality rate, milder hospital outcomes, and lower rates of early hospital readmissions in obese patients with TAK. Perhaps, increased sympathetic nervous system drive in the population with normal weight may explain these findings [14].

In the CAD and PAD populations, the obesity paradox was not observed. The prevalence of CAD and PAD increased significantly in the overweight and obese groups. The PAD population was 12.1% in the HW group compared to 16.7% in the OB3 group, and the CAD population was 18.7% in the HW group compared to 25% in the OB1 group. Another study revealed that obesity increased the odds of PAD. Their study also notes that severe PAD leading to chronic limb ischemia may be higher in the obese population. They postulated that these findings might be due to the pro-inflammatory state caused by obesity. High adipocyte levels have been linked with increased circulating IL-6, TNF-α, and several cytokines [6,14]. Another explanation is that obesity alters fibrinolysis and coagulation cascade leading to disruption of the microcirculation, which leads to hypercoagulability that favors PAD, CAD, STEMI, and NSTEMI [6,14]. Also, hypertension, dyslipidemia, and diabetes are common in the population with obesity, contributing to PAD progression and severity. A study of 3,155,387 sex-stratified populations showed that the prevalence of PAD was higher in the obese population, with a higher proportion in the female gender. The prevalence to BMI ratio in women was revealed as a J-shaped curve. However, in the male population, there was a greater prevalence of PAD in underweight and healthy weight groups. The correlation between PAD and obesity was only seen in the group with a BMI > 40 kg/m2 [15].

Previous studies have shown that BMI was associated with an overall increased cardiovascular morbidity and mortality but inconsistent across the spectrum of cardiovascular disease [15,16]. Das et al. used the National Cardiovascular Data Registry (NCDR) to demonstrate that although individuals with class three obesity presenting with a STEMI were, on average, younger, they had a higher prevalence of diabetes, hypertension, dyslipidemia, a less extensive CAD, and better ejection fraction; they still had a higher in-hospital mortality despite their advantages [15]. This was somewhat similar to our study, although our outcomes of interest were mainly cardiovascular interventions with little emphasis on in-hospital complications. In our study, the mean age of the population with obesity was 61 years (SD = 16) in the OB1 group, 58 years (SD = 15.9) in the OB2 group, and 55 years (SD = 15.5) in the OB3 group. The mean ages in the HW and OV groups were 68 years (SD = 16.6) and 65 years (SD = 16.1), respectively. With regard to mortality, the HW group that underwent PCI had a mortality of 6.61% (p < 0.0001). Still, the result was inconsistent amongst the OV and obese group, with the OV group having a PCI mortality rate of 1.77% (p = 0.08), the OB1 group having a mortality rate of 1.42% (p < 0.0001), the OB2 group having a mortality rate of 1.59% (p < 0.0001), and the OB3 group having a mortality rate of 2.29% (p = 0.08). This may be supported by other studies that have found an obesity paradox in individuals with obesity undergoing PCI. Angerås et al. demonstrated that of the 64,436 patients undergoing PCI, the obese and overweight populations had the least mortality compared to the normal-weight individuals; however, they also found that those with morbid obesity had higher mortality [16]. Younge et al. also showed that amongst 1019 patients who underwent PCI, the seven-year mortality was reduced in the overweight population but not in the obese population [17]. In a retrospective analysis conducted by Lazzeri et al., they analyzed the one-year STEMI outcomes in patients who received PCI. This population was divided into lean, normal, overweight, and obese according to BMI. After a one-year follow-up, they concluded that the population with a lean BMI had the highest mortality risk regardless of age. They also noted that the younger population with obesity (<75 years) had the lowest mortality, albeit at short-term follow-up [18].

Explaining the obesity paradox

**Figure 2:**
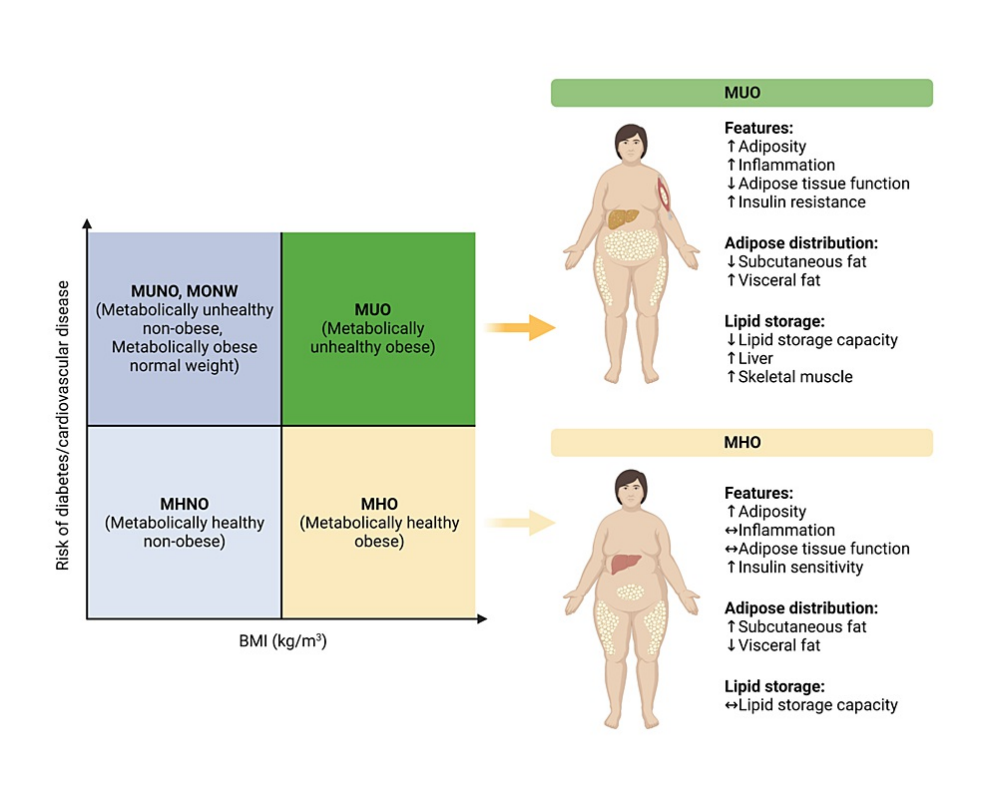
Obesity phenotype Original illustration created with BioRender.com.

Hypothetically, an increase in BMI leads to a decent increase in the size of coronary arteries. This may suggest why individuals with obesity have better PCI outcomes since narrower coronaries are associated with worse outcomes after PCI [19-21]. Some studies suggest that the hormonal and cytokines profile is altered in obesity and may be cardio-protective by neutralizing upregulated factors in acute or chronic heart disease, such as the neutralization of inflammatory TNF-α by high-density TNF-α receptors on the adipocytes [9,14,22]. Furthermore, individuals with obesity have been shown to have lower levels of a natriuretic peptide that is linked to the pathophysiology of heart failure [21,23]. Obese individuals are protected against malnutrition because they have a higher store of calories at baseline. Hence, cardiac remodeling is greater following infarction and is protected from cardiovascular disease-induced cachexia, which confers a worsening prognosis [24]. Figure 2 below delineates the obesity phenotype and how it may impact the outcomes of cardiovascular events by categorizing the obese population into metabolically unhealthy obese (MUO) and metabolically healthy obese (MHO) population.

Shifting the paradigm to prioritize obesity management in cardiovascular health

The weight loss and lifestyle modification approach has evolved over the years. Recent groundbreaking science in weight management needs to be harnessed extensively to benefit the large population with cardiovascular disease and risk factors [24-26]. The first consideration would be thoroughly reviewing their medical history, medication regimen, and pill burden. The baseline approach is to encourage patients to try to expend more energy than they consume. This, however, is not feasible in a handful of elderly or disabled patients with multiple comorbidities. A general overview of the health benefits of daily habits, including healthy eating and increased physical activity, helps individuals with some mental picture for their future endeavors [24-27]. The discussion with patients about weight loss must be conversational and void of judgmental insinuations or tone. This is important for the physician-patient relationship.

Tailoring the weight loss need of patients based on their comorbidities, baseline activity level, and overall health status is crucial. For individuals with type 2 diabetes mellitus (T2DM), medications such as glucagon-like peptide-1 (GLP-1) agonists that may not interfere with blood glucose levels and thus not require medication adjustments may be preferred. The GLP-1 helps weight loss by slowing down the gut and decreasing the sensation of hunger. Patients feel full much sooner and can achieve significant weight loss over time. In patients with heart failure (HF), sodium-glucose transporter 2 (SGLT-2) inhibitors may be preferred. They result in modest weight loss through renal excretion of sodium. Their impact on cardiac function is remarkable as they have mortality-reducing effects. Understanding what the patients deem successful weight loss is always a great approach. This can give a perception of how aggressive or long your consideration for some interventions may be [25-29]. This paradigm is important to our study because we highlight the increased prevalence of cardiovascular interventions in the obese population. Perhaps active weight management and nutrition lifestyle changes should be enforced among the target population from hospitalization to ensure maximal benefit to this population.

Strengths and limitations of the study

This study uses one of the largest available inpatient samples of the US population. A sample of over 5.6 million hospitalizations was stratified according to BMI. The NIS database is useful for research, albeit designed as an administrative tool for billing purposes, and relies on the coders' accuracy, hence the possibility of overbilling, underbilling, and wrong coding. Also, we did not include baseline characteristics of patients or confounding factors since we only conducted a prevalence study, not a comparative one. The obesity phenotype is crucial in predicting cardiovascular outcomes. However, the NIS cannot differentiate between obesity phenotypes besides the assigned BMIs. Some missing frequencies may impact the analysis. The NIS cannot differentiate between multiple hospitalizations from a single patient, hence it can result in duplication.

## Conclusions

Our study supports the evidence of increased cardiovascular interventions in direct proportion to increasing BMI. Albeit, an inconsistent presentation across the spectrum of cardiovascular diseases and outcomes, for example, equal or better outcomes in obese cohorts compared to the healthy-weight population undergoing PCI. However, the increasing cardiovascular intervention burden in the youngest studied population suggests a rise in the cardiovascular disease burden among the young and partially explains their better outcomes. There is a tendency that counseling patients about weight loss may be done passively. Active steps are needed to get these patients from the cardiologist to the weight management specialist. Perhaps organizing the electronic medical records system such that it includes an automatic consultation with the weight management specialist for every overweight or obese individual with cardiovascular disease or risk factors should be considered a routine.
